# Analysis of *ERBB4* Variants in Amyotrophic Lateral Sclerosis Within a Chinese Cohort

**DOI:** 10.3389/fneur.2022.865264

**Published:** 2022-04-11

**Authors:** Fan Wang, Xiangyi Liu, Ji He, Nan Zhang, Lu Chen, Lu Tang, Dongsheng Fan

**Affiliations:** ^1^Department of Neurology, Peking University Third Hospital, Beijing, China; ^2^Beijing Municipal Key Laboratory of Biomarker and Translational Research in Neurodegenerative Diseases, Beijing, China; ^3^Key Laboratory for Neuroscience, National Health Commission/Ministry of Education, Peking University, Beijing, China

**Keywords:** China, amyotrophic lateral sclerosis, *ERBB4*, variant, clinical features

## Abstract

*ERBB4* is related to amyotrophic lateral sclerosis (ALS) in patients with a family history and is thought to cause ALS-19. We screened 448 ALS patients, including 364 sporadic ALS (sALS) and 84 familial ALS (fALS) patients with *ERBB4* variants, in a Chinese cohort. In total, 12 missense variants were identified in this study. Of these, 3 (p.Arg106His, p.Gln164Pro, and p.Val212Leu) were absent from the in-house healthy control cohort and population databases and predicted to be likely pathogenic. Genetic burden analysis did not reveal an increase in damaging variants of the *ERBB4* gene. We considered that most of the missense variants in *ERBB4* were not pathogenic, but certain variants, such as p.Arg106His, p.Gln164Pro, and p.Val212Leu, were likely pathogenic. The phenotype of these three patients carrying *ERBB4* variants revealed the typical clinical manifestations of ALS without cognitive dysfunction. We concluded that *ERBB4* likely pathogenic variants account for ~0.67% of ALS patients in China. It is necessary to interpret the relationship between the disease and variants carefully for ALS patients with *ERBB4* gene variants.

## Introduction

Amyotrophic lateral sclerosis (ALS) is a progressive and fatal neurodegenerative disease that mainly affects the upper and lower motor neurons of the spinal cord and brain (1). The ALS incidence in European populations has been estimated to be 2.16 cases per 100,000 people per year, and it usually leads to death ~3–4 years after onset (2, 3). In the Chinese population, the ALS incidence is 1.65 per 100,000 people per year (4), and the median survival time is 71 months after symptom onset (5). Furthermore, the clinical characteristics seem to be different between Chinese ALS patients and ALS patients in Caucasian population (6).

Approximately 5–10% of patients have a positive family history (fALS), while the remaining cases are sporadic (sALS). Interestingly, the gene mutations of fALS are also present in sALS. Genetic factors play an important role in the pathogenesis of ALS (7). The genetic cause of ~10% of sALS and 2/3 of fALS in the Caucasian population has been determined (8). Importantly, there is likely to be genetic heterogeneity between ethnic groups with ALS (9). Our previous studies have shown that the frequency of gene variant differences between Chinese and Caucasian ALS patients could be a primary reason underlying the distinct clinical features (10, 11). To date, more than 25 mutant genes have been shown to cause or significantly increase the risk of ALS.

*ERBB4* is a transmembrane tyrosine kinase of the epidermal growth factor receptor (EGFR) family that includes *ERBB1, ERBB2, ERBB3, and ERBB4* (12). After binding of neuregulin (NRG) to other members of the subfamily, such as *ERBB2* or *ERBB3, ERBB4* forms homodimers or heterodimers to activate the C-terminal domains and autophosphorylation of its tyrosine kinase, thereby mediating various downstream signaling cascades (13). *ERBB4* plays an essential role in neurodevelopment, such as nerve conduction and synaptic plasticity, and it is also related to the occurrence of mental illness and epilepsy (14, 15). The *ERBB4* mutation was first found in a family of ALS patients in 2013 and was believed to be a new pathogenic gene of ALS-19 (16). A study conducted a genetic analysis of known ALS/FTD patients that did not carry the *C9orf72* expansion mutation and found that the *ERBB4* mutation (c.1997T>C, p.Ile666Thr) was the probable pathogenic variant (17). A heterozygous mutation in *ERBB4* (c.2136T>G, p.Ile712Met) was also reported in late-onset alS/FTD (18). Moreover, in the pathophysiology of ALS, immunofluorescence staining showed that *ERBB4* immunoreactivity was decreased in the spinal cord of sALS patients, which further indicates that *ERBB4* is involved in the pathophysiological process of sALS (19). In this study, we screened *ERBB4* variants in a large cohort of ALS patients from mainland China to determine their pathogenicity and frequency.

## Materials and Methods

### Subjects

This study included 448 Chinese ALS patients who were admitted to the Department of Neurology, Peking University Third Hospital from January 2015 to July 2021. All patients were diagnosed with definite, probable, or laboratory-supported probable ALS according to the revised El Escorial criteria (20), and patients who had ALS-like syndrome caused by paraneoplastic or autoimmune diseases or with suspected or possible ALS were excluded. A total of 1,812 control subjects without any neurological disease history were included in the in-house control cohort. Written informed consent for genetic analysis was obtained from all participants. This study was approved by the ethics committee at Peking University Third Hospital (IRB No. 00006761).

### Genetic Studies

According to standard procedures, patient and control genomic DNA was extracted from peripheral blood samples (Qiagen, Valencia, California). DNA was analyzed predominantly by targeted next-generation sequencing (NGS), which was carried out at Kangso Medical Inspection Company (Beijing, China) following standard experimental protocols. The panel of ALS related gene tested in this study were list in Supplementary Table 1. Sequencing reactions were designed to include all coding regions, as well as the flanking ~100 bp of intronic DNA for each exon and 3′ and 5′ untranslated regions (UTRs) of the *ERBB4* gene (NM_005235). The quality of the sequencing data was assessed using the BWA, Samtools, Picard, and Genome Analysis Toolkit (GATK) available from Babraham Bioinformatics (http://www.bioinformatics.babraham.ac.uk/). Assessing factors included the allele balance, coverage uniformity, the total count of reads, the percentage of reads that matched the sequence of the human genome and were located inside the target sequence of the target gene, and the average sequencing depth. DNASTAR Lasergene v7.1 was used to compare the cDNA sequence with the reference *ERBB4* mRNA sequence. The *ERBB4* variants were compared with those reported in the Single Nucleotide Polymorphism Database (dbSNP; https://www.ncbi.nlm.nih.gov/snp), ChinaMAP (http://www.mbiobank.com/), gnomAD (East Asian, EA) (https://gnomad.broadinstitute.org) and 1000 Genomes Projects (East Asian, EA) (http://browser.1000genomes.org) databases.

*In silico* tools were applied to identify the functional effects of the *ERBB4* missense variants, including PolyPhen2 (http://genetics.bwh.harvard.edu/pph2/), SIFT (http://sift.jcvi.org), Mutation Taster (http://www.mutationtaster.org), and CADD. Sequence homologs were aligned to analyze the level of evolutionary conservation using the UniProt website (http://www.uniprot.org/). Three methods were used for the burden analysis of the rare variants of the *ERBB4* gene, including the C-alpha test, the Rare-Variant Weighted Aggregate Statistic (RWAS) and the Sequence Kernel Association Test (SKAT), using the R package *AssotesteR*. Two levels of rare variants were evaluated: (1) rare exon variants with minor allele frequency (MAF) <1% in the population database (gnomAD) (the rare variants set); (2) ultrarare missense variants (presented less than twice in the gnomAD database, according to the prevalence of ALS) with Combined Annotation Dependent Depletion (CADD) score>20 (the ultrarare variants set). The *p*-values of 10,000 resamplings were also reported. Suspected variants were interpreted mainly based on the American College of Medical Genetics and Genomics (ACMG) guidelines. The structures of wild-type and mutated *ERBB4* were calculated according to their alignment by SWISS-MODEL Server (Swiss Institute of Bioinformatics, Lausanne, Switzerland) (21). Graphics were generated using Swiss-Pdb viewer 3.7 software after put the structures of wild-type and mutated *ERBB4* into it (22). The *ERBB4* protein domain was derived from the National Center for Biotechnology Information (NCBI, https://www.ncbi.nlm.nih.gov) after searched with ERBB4 as a keyword in NCBI. All cases with potentially pathogenic variants in *ERBB4* were also sequenced for *TARDBP, FUS, SOD1*, and *C9ORF72*.

## Results

### Cohort Description

In total, 448 participants with ALS and 1,812 healthy control subjects were screened. The proportions of definite, probable, or laboratory-supported probable ALS patients were 18.38, 39.46, and 42.16%, respectively. Among the patients, 84 patients were unrelated fALS probands, and 364 patients were considered to have sALS. In total, 293 patients were males, and the remaining 155 patients were females. The mean age of onset ± SD was 42 ± 11.31 years old.

### Genetic Analysis

In this study, the total count of reads was 8-9 G, and the percentage of reads that matched the human genome sequence was ~99.9%. The percentage of reads that were located inside the target sequence of the *ERBB4* gene was above 99.8%, and the average sequencing depth was above 100 × .

Twelve missense variants were identified in 16 unrelated ALS patients, and their allele frequencies in the healthy control cohort and the public population databases are listed in Table 1. Neither small insertion and deletion mutations nor splice site mutations were found. The variants were all heterozygous. All 12 missense variants were rare variants that were defined as heterozygous variants in dominant ALS-causative genes with MAF <0.1% in any of the following databases: ChinaMAP, gnomAD (EA) and 1000 Genomes Projects (EA). We also identified 278 non-synonymous variants in the control cohort (Supplementary Table 2).

**Table 1:** *ERBB4* missense variants identified both in ALS patients and control subjects with related information in the public database.

**Chr:Position (GRCh37)**	**cDNA change**	**Protein change**	**dbSNP**	**Exon**	**Frequency in patient allele**	**Frequency in control allele**	**China map**	**Gnome AD (EA)**	**1,000G (EA)**
2:212812292	c.284G>A	p.Arg95His	rs778048381	3	1/448	1/1,812	10/21,176	8/18,964	0
2:212812259	c.317G>A	p.Arg106His	rs991337964	3	1/448	0/1,812	0	0	0
2:212652815	c.491A>C	p.Gln164Pro	Novel	4	1/448	0/1,812	0	0	0
2:212589908	c.634G>T	p.Val212Leu	Novel	6	1/448	0/1,812	0	0	0
2:212495294	c.1972A>T	p.Ile658Phe	rs190654033	17	5/448	20/1,812	123/21,176	68/19,952	0.003
2:212495266	c.2000T>C	p.Val667Ala	rs138313493	17	1/448	0/1,812	0	0	0
2:212293145	c.2707G>A	p.Val903Ile	rs764667767	22	1/448	0/1,812	3/21,176	0	0
2:212285326	c.2975G>A	p.Arg992His	rs1390491269	25	1/448	0/1,812	0	2/18,374	0
2:212252710	c.3143T>C	p.Ile1048Thr	rs1222680082	26	1/448	0/1,812	0	2/19,952	0
2:212251704	c.3355G>A	p.Val1119Ile	rs1217217544	27	1/448	0/1,812	0	1/18,392	0
2:212248613	c.3654A>T	p.Lys1218Asn	Novel	28	1/448	0/1,812	0	0	0
2:212248498	c.3769G>C	p.Asp1257His	rs766270456	28	1/448	1/1,812	3/21,176	13/18,376	0

**Figure 1:**
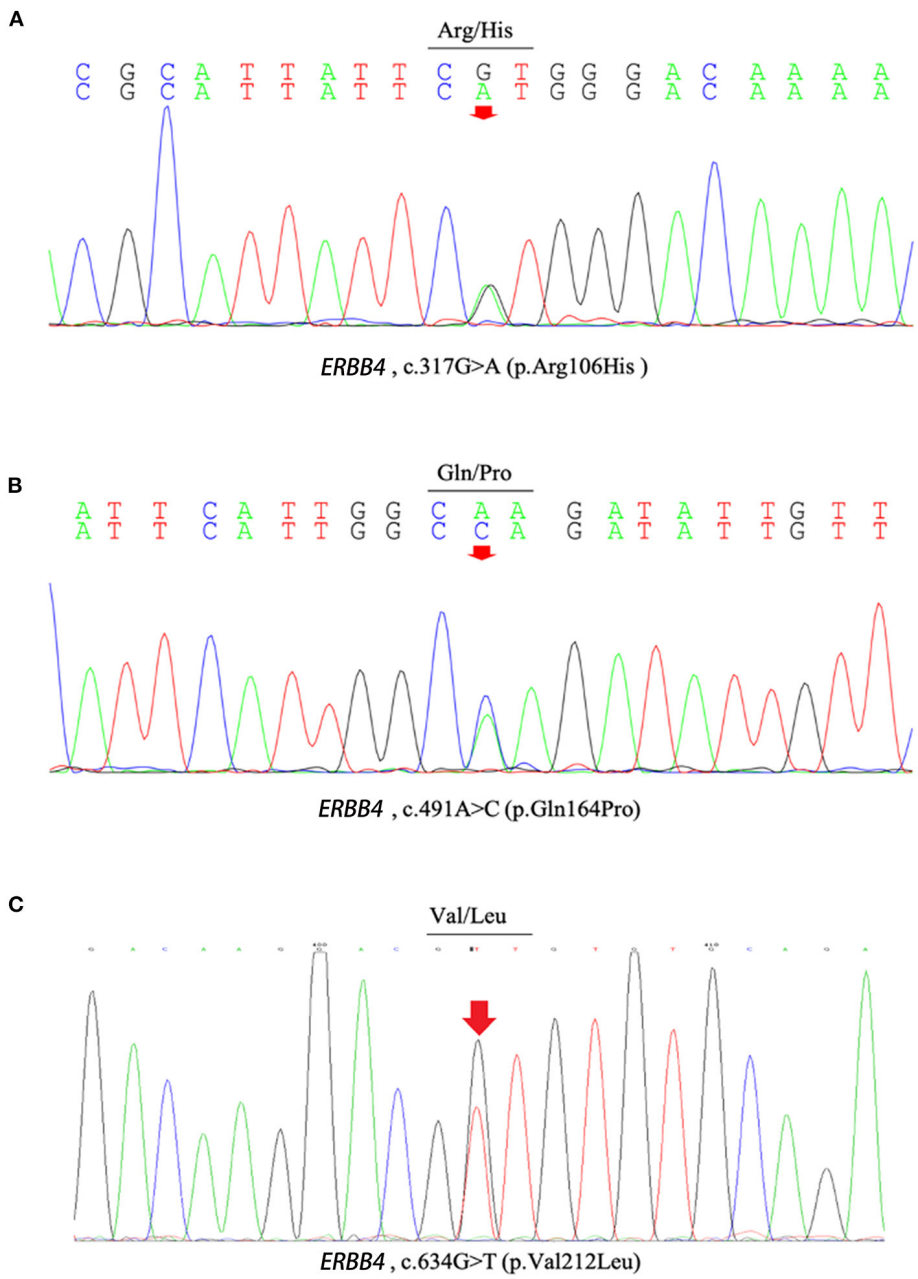
The chromatograph of *ERBB4* variants (p.Arg106His, p.Gln164Pro and p.Val212Leu). **(A)** The chromatograph of *ERBB4* p.Arg106His variant. **(B)** The chromatograph of *ERBB4* p.Gln164Pro variant. **(C)** The chromatograph of *ERBB4* p.Val212Leu variant.

*Key: dbSNP, The Single Nucleotide Polymorphism Database; gnomAD, Genome Aggregation Database; ChinaMAP, China Metabolic Analytics Project*.

c.317G>A (p.Arg106His), c.491A>C (p.Gln164Pro), c.634G>T (p.Val212Leu), and c.2000T>C (p.Val667Ala), c.3654A>T (p.Lys1218Asn) were present neither in the control cohort nor in any of the public population databases, including ChinaMAP, gnomAD (EA), 1000 Genomes Projects (EA) and dbSNP. The functional effects of these 5 missense variants were predicted by PolyPhen2, SIFT, Mutation Taster, and CADD (Table 2). The functional effects of c.2000T>C (p.Val667Ala) and c.3654A>T (p.Lys1218Asn) were benign predicted by *in silico* tools. The patients who carried other 3 variants (p.Arg106His, p.Gln164Pro and p.Val212Leu) did not carry common pathogenic mutations related to ALS, such as mutations in *SOD1, TARDBP, FUS*, and *C9ORF72* (Supplementary Figure 1). Sequences of the 3 variant sites were validated by Sanger sequencing (Figure 1).

**Table 2:** Results predicted by *in silico* tools of the missense variants which were present neither in control cohort nor in any of the population polymorphism databases.

**cDNA change**	**Amino-acid change**	**SIFT**	**Polyphen2**	**MutationTaster**	**CADD**
**c.317G>A**	**p.Arg106His**	**Deleterious**	**Deleterious**	**disease_causing**	**31**
**c.491A>C**	**p.Gln164Pro**	**Deleterious**	**Benign**	**disease_causing**	**23.4**
**c.634G>T**	**p.Val212Leu**	**Tolerated**	**Probably damaging**	**disease_causing**	**24.6**
c.2000T>C	p.Val667Ala	Tolerated	Benign	polymorphism	21
c.3654A>T	p.Lys1218Asn	Tolerated	Tolerated	polymorphism	0.001

*The 3 variants (p.Arg106His, p.Gln164Pro, and p.Val212Leu) in bold were likely to be pathogenic*.

### Clinical Features of Patients With *ERBB4* Mutations

**Figure 2:**
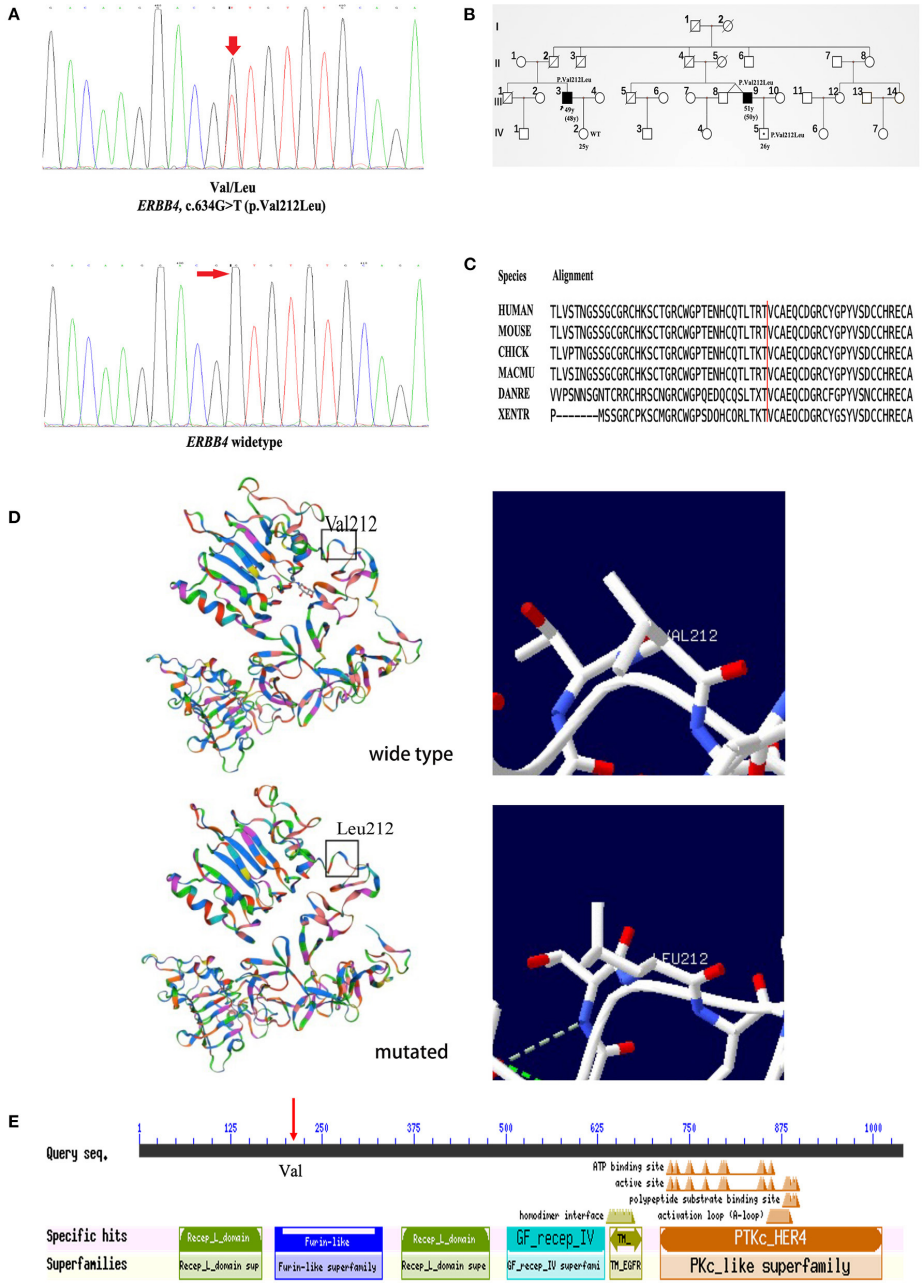
Genetic analysis of the pedigree carrying the *ERBB4* p.Val212Leu mutation. **(A)** The chromatograph of *ERBB4* p.Val212Leu mutation and its normal control. **(B)** Pedigree structure of family carrying the *ERBB4* p.Val212Leu mutation (III3 and III9: Affected person carrying mutant gene. IV5: Unaffected person carrying mutant gene). **(C)** The *ERBB4* p.Val212Leu residue is highly conserved across multiple species. **(D)** The structures of wild-type *ERBB4* and mutated *ERBB4*. **(E)** The *ERBB4* protein domain and the mutation site were located in the Furin-like cysteine-rich region (red arrow).

Clinical information of the 16 unrelated ALS patients with 12 missense variants are list in Table 3. All three patients carrying c.317G>A (p.Arg106His), c.491A>C (p.Gln164Pro), and c.634G>T (p.Val212Leu) were males. The average onset age was 49.33 ± 7.10 years old. Two patients had limb onset, and one patient had bulbar onset. However, none of these patients had cognitive dysfunction. They were all alive, and their survival times were 27, 30, and 60 months. The patient with the c.317G>A (p.Arg106His) variant had limb onset at the age of 57 years old. He felt weakness in his left foot in 2016, and his condition gradually worsened. He had left foot drops and unsteady walking 2 years later. By 2020, he developed muscle atrophy in his left leg and weakness in his right hand. The patient with the c.491A>C (p.Gln164Pro) variant had limb onset at the age of 43 years old. In May 2019, the patient developed left arm weakness, muscle atrophy, and fasciculations, and his condition gradually worsened too. Seven months later, he was diagnosed with probable ALS. The patient with the c.634G>T (p.Val212Leu) variant had bulbar onset at the age of 48 years, and 18 months later, he was diagnosed with probable ALS. Initially, he mainly suffered from slurred speech, choking on drinking water, and difficulty swallowing in February 2019. Later, he developed finger weakness and difficulty lifting his upper limbs, which was accompanied by fasciculations. Unfortunately, he was a familial patient, and his cousin had similar symptoms and the same mutation site as him. His cousin's son also carried the same mutation at this locus but did not show any clinical symptoms. Interestingly, his cousin's twin brother had no similar symptoms. The chromatograph of *ERBB4* p.Val212Leu mutation and its normal control is shown in Figure 2A. The pedigree of this family is shown in Figure 2B. The Val212 residue was highly conserved across different species (Figure 2C). The structures of wild-type *ERBB4* and mutated *ERBB4* were calculated according to their alignment (Figure 2D). As shown in the schematic of the *ERBB4* protein domain, the mutation site was located in the Furin-like cysteine-rich region (shown by the red arrow, Figure 2E).

**Table 3:** Clinical information of the 16 unrelated ALS patients with 12 missense variants.

**Variant**	**Sex**	**Age at onset (y)**	**Site of onset**	**Survival time (m)**	**Cognitive impairment**	**Family history**
p.Arg95His	Female	41	Spinal (arm)	22^c^	No	No
**p.Arg106His**	**Male**	**57**	**Spinal (leg)**	**60** ^c^	**No**	**No**
**p.Gln164Pro**	**Male**	**43**	**Spinal (arm)**	**27** ^c^	**No**	**No**
**p.Val212Leu**	**Male**	**48**	**Bulbar**	**30** ^c^	**No**	**Yes**
p.Ile658Phe	Female	57	Bulbar	NA	No	No
p.Ile658Phe	Female	50	Spinal (leg)	249^c^	No	Yes
p.Ile658Phe	Male	35	Spinal (leg)	15^c^	No	No
p.Ile658Phe	Female	44	Spinal (leg)	54^c^	No	No
p.Ile658Phe	Female	55	Spinal (arm)	20^a^	Yes	No
p.Val667Ala	Male	36	Spinal (leg)	169^c^	No	Yes
p.Val903Ile	Male	39	Spinal (leg)	16^b^	No	Yes
p.Arg992His	Male	46	Spinal (arm)	26^c^	No	No
p.Ile1048Thr	Male	40	NA	NA	No	Yes
p.Val1119Ile	Female	37	Spinal (arm)	62^c^	No	No
p.Lys1218Asn	Male	62	Spinal (leg)	20^c^	No	No
p.Asp1257His	Male	36	Spinal (arm)	23^c^	No	No

*NA, Not available*.

a *Dead*.

b *Tracheotomy state*.

c *Alive*.

*The 3 variants (p.Arg106His, p.Gln164Pro, and p.Val212Leu) in bold were likely to be pathogenic*.

### Burden Analysis

The results of burden analysis performed by three methods (C-alpha, RWAS, SKAT) are listed in Table 4. A total of 448 ALS patients and 1,812 neurologically normal control individuals were included in the analysis. There was no significant difference between the two variant sets.

**Table 4:** Results of burden tests in ALS patients and controls.

	**Rare variants set**		**Ultrarare variants set**	
	***p*-value**	**Resampling *p*-value**	***p*-value**	**Resampling *p*-value**
C-alpha Test	0.148	0.078	0.004	0.003
RWAS	0.981	0.999	0.998	0.999
SKAT	0.956	0.996	0.999	0.999

## Discussion

Here, 448 participants with ALS and 1,812 healthy control subjects were screened. Among them, 84 patients were unrelated fALS probands, and 364 patients were considered to have sALS. We identified 12 missense variants in 16 unrelated ALS patients, and this was the first report of these variants in ALS patients, except for c.284G>A (p.Arg95His) (23). There were 3 variants (p.Arg106His, p.Gln164Pro, and p.Val212Leu) that were likely to be pathogenic, especially the p.Val212Leu variant. The incidence of likely pathogenic variants in our ALS cohort was 0.67% (3/448), and that in the fALS cohort and sALS cohort were 1.19% (1/84) and 0.55% (2/364), respectively. However, the incidence of *ERBB4* mutations in sALS was 0.29% (2/691) in central South China (24). This was lower than that in the present study and might be due to the different screening criteria used. In other studies of the genetic spectrum and variability in ALS patients, researchers found several *ERBB4* variants, such as p.Met322Lys (25), Glu69Val, Arg103His (26, 27), p.Gly1272Arg, p.His374Gln and p.Met1059Thr, and the p.Gly1272Arg variant was a probable pathogenic variant (28).

The p.Val212Leu variant was found in fALS. The Val212 residue is highly conserved across different species, and high evolutionary conservation always suggests the functional importance of a position. Several lines of evidence support the pathogenicity of the p.Val212Leu variant. First, the variant was located in the well-studied functional domain without benign variation (PM1). Second, the variant was not reported in any of the public databases, such as dbSNP, ChinaMAP, gnomAD (EA) and 1000 Genomes Projects (EA) (PM2). Third, the variant cosegregated with the phenotype of ALS in the affected family members (PP1). Furthermore, this variant was classified as disease-causing by several *in silico* predictions, and the Val212 residue was highly conserved across species (PP3). Last, the patients' clinical phenotype in this family was highly consistent with the disease caused by the *ERBB4* variant (PP4). Therefore, according to the ACMG variant classification criteria, the p.Val212Leu variant may be classified as “likely pathogenic” (PM1+PM2+PP1+PP3+PP4) (29). Combining the functional prediction results of the *in silico* tools with a CADD score>20, we also inferred that the c.317G>A (p.Arg106His), c.491A>C (p.Gln164Pro), and c.634G>T (p.Val212Leu) variants were likely to be pathogenic.

The mutation region was in the Furin-like cysteine-rich region of the *ERBB4* protein. Studies have proven that the Furin-like cysteine-rich region mainly mediates the Wnt/β-catenin signaling pathway, which plays a potential role in the pathogenesis of ALS (30–33). The abnormal activation of this pathway is related to neuronal degeneration and glial cell proliferation (33). Two previous studies found that the pathogenesis of *ERBB4* mutations in ALS was related to decreased autophosphorylation (16, 18). According to the structure prediction, there was no obvious differences between mutant and wildtype protein. Previous study has shown that the RMSD/RMSF plot is helpful in determining the effect of amino acid changes on protein structure (34), other methods including mRNA expression level and animal models are also needed to verify the pathogenicity of p.Val212Leu in the future.

Although we considered the p.Arg106His, p.Gln164Pro, and p.Val212Leu variants were likely pathogenic, the burden analysis showed that the rare variants in our study were not significantly different from the controls. It was indicated that most of the missense variants in *ERBB4* were not pathogenic, but certain variants, such as p.Arg106His, p.Gln164Pro, and p.Val212Leu, were likely pathogenic. Therefore, it is necessary to interpret the relationship between the disease and variants carefully for ALS patients with *ERBB4* gene variants.

In our study, clinical information of the three likely pathogenic variants (p.Arg106His, p.Gln164Pro, and p.Val212Leu) revealed that all three patients were males, and the average age at onset was 49.33 ± 7.10 years old. The ratio of bulbar onset to limb onset was 1:2, and none of these patients had cognitive dysfunction. A study of 11 ALS patients with *ERBB4* variants in southern China demonstrated similar clinical characteristics to our present study. The male/female ratio was 9:2, and the average onset age was 49.5 ± 12.6 years old. The limb-onset/bulbar-onset ratio was 10:1, and none of these patients had cognitive dysfunction (23). In our previous study on the clinical characteristics of ALS patients from 2015 to 2018, it was shown that the male/female ratio was 1.85:1, the average onset age was 53.0 ± 11 years old, and the limb-onset/bulbar-onset ratio was 3.71:1 (6). Similar to our study, most previous reports have shown that ALS patients with *ERBB4* variants did not have cognitive dysfunction, except for two heterozygous variants of *ERBB4* (c.2136T>G, p.Ile712Met; c.1997T>C, p.Ile666Thr) (17, 18).

This study has certain limitations. Due to some special reasons, such as the impact of the new coronavirus epidemic, our collection of the data on ALS families was not comprehensive, which affected our judgment on the pathogenicity of the mutation. Long-term follow-up observation of the families is needed. In addition, because ALS is both clinically and genetically a highly heterogeneous disease (35), it may reduce the power to detect some effects. Furthermore, all patients who participated in the research were recruited from China. Due to the long history of intermarriage among the population, the patient population in this study was heterogeneous, which may further limit our analysis power.

## Conclusions

In this Chinese ALS cohort, 12 missense variants were identified, and 3 of these variants (p.Arg106His, p.Gln164Pro, and p.Val212Leu) were likely pathogenic. We considered that most of the missense variants in *ERBB4* were not pathogenic, but certain variants, such as p.Arg106His, p.Gln164Pro, and p.Val212Leu, were likely pathogenic, especially the p.Val212Leu variant. We concluded that the incidence of likely pathogenic variants in our ALS cohort was 0.67% (3/448), and that of the fALS cohort and sALS cohort were 1.19% (1/84) and 0.55% (2/364), respectively. Therefore, it is necessary to interpret the relationship between the disease and variants carefully for ALS patients with *ERBB4* gene variants.

## Data Availability Statement

The data presented in the study are deposited in the NCBI SRA repository, accession number PRJNA814828, the project information is accessible with the following link: http://www.ncbi.nlm.nih.gov/bioproject/814828.

## Ethics Statement

The studies involving human participants were reviewed and approved by Ethics Committee at Peking University Third Hospital. The patients/participants provided their written informed consent to participate in this study. Written informed consent was obtained from the individual(s) for the publication of any potentially identifiable images or data included in this article.

## Author Contributions

FW, XL, and DF conceived the study. FW and XL performed the experiments and data analyses. JH, NZ, LT, and LC conducted the patient enrolment and follow-up. FW wrote the manuscript. XL and DF revised the manuscript. All authors contributed to the article and approved the submitted version.

## Funding

This study was supported by Grants from the National Natural Science Foundation of China (Grant Numbers 81873784, 82071426, 82001361, and 81974197), Clinical Cohort Construction Program of Peking University Third Hospital (Grant Number BYSYDL2019002).

## Conflict of Interest

The authors declare that the research was conducted in the absence of any commercial or financial relationships that could be construed as a potential conflict of interest.

## Publisher's Note

All claims expressed in this article are solely those of the authors and do not necessarily represent those of their affiliated organizations, or those of the publisher, the editors and the reviewers. Any product that may be evaluated in this article, or claim that may be made by its manufacturer, is not guaranteed or endorsed by the publisher.
