# Supplementary material for: Analysis of ERBB4 Variants in Amyotrophic Lateral Sclerosis Within a Chinese Cohort
Source: Front Neurol. 2022 Apr 11;13:865264. doi: 10.3389/fneur.2022.865264 (PMC9035935; doi:10.3389/fneur.2022.865264)
Supplement: Supplementary file 1 [file Data_Sheet_1.PDF]

Analysis of *ERBB4* variants in amyotrophic lateral sclerosis within a Chinese cohort

Supplementary Material

Supplementary table 1. The panel of ALS related gene tested in this study.

AAAS	*AARS1*	*ABCA1*	*ABCA2*	*ABCD1*	*ABHD12*	*ACSM3*
ADAR	*AFG3L2*	*AGXT*	*AIFM1*	*AK9*	*ALAD*	*ALDH18A1*
ALS2	*AMACR*	*AMPD2*	*ANAPC7*	*ANG*	*ANXA11*	*AP4B1*
AP4E1	*AP4M1*	*AP4S1*	*AP5Z1*	*APEX1*	*APOE*	*APTX*
AR	*ARG1*	*ARHFEF10*	*ARHFEF28*	*ARL6IP1*	*ARSA*	*ARSI*
ATL1	*ATL3*	*ATP13A2*	*ATP2B4*	*ATP7A*	*ATP7B*	*ATP8B3*
ATRX	*ATXN1*	*ATXN2*	*ATXN3*	*B4GALNT1*	*BAG3*	*BCL11B*
BCL6	*BCL7A*	*BICD2*	*BSCL2*	*C19orf12*	*C6*	*C9orf72*
CABIN1	*CACNA1H*	*CAMK1G*	*CAPN1*	*CCL5*	*CCNF*	*CCR2*
CCR5	*CCT5*	*CD33*	*CD59*	*CDH1*	*CDH13*	*CDH22*
CFAP410	*CHCHD10*	*CHCHD2*	*CHGB*	*CHMP1B*	*CHMB2B*	*CHRM1*
CHRNA4	*CLEC4C*	*CNGA4*	*CNOT1*	*CNTN6*	*CNTNAP2*	*COASY*
COL19A1	*COL7A1*	*COQ4*	*COQ7*	*COX6A1*	*CP*	*CPOX*
CPT1C	*CRIM1*	*CRTC1*	*CRYM*	*CTDP1*	*CTSD*	*CX3CR1*
CYP27A1	*CYP2C9*	*CYP2E1*	*CYP2U1*	*CYP7B1*	*DAO*	*DARS2*
DAZ1	*DAZL*	*DCAF17*	*DCAF8*	*DCTN1*	*DCTN2*	*DDHD1*
DDHD2	*DENND2C*	*DGAT2*	*DHFR*	*DHH*	*DHTKD1*	*DIAPH3*
DMD	*DMXL2*	*DNAH10*	*DNAH2*	*DNAH9*	*DNAJB2*	*DNM2*
DNMT1	*DOC2B*	*DPP6*	*DPYSL3*	*DRP2*	*DST*	*DSTYK*
DYNC1H1	*EGR2*	*EIF4E1B*	*ELP3*	*ENO1*	*ENTPD1*	*EPHA4*
ERBB4	*ERLIN1*	*ERLIN2*	*EWSR1*	*EXOSC3*	*FA2H*	*FARS2*
FBLN5	*FBXO38*	*FCGR3B*	*FEZF2*	*FGD4*	*FGF14*	*FGFR4*
FGGY	*FIG4*	*FLNC*	*FLRT1*	*FMR1*	*FOXN3*	*FTL*
FUS	*FXN*	*GALC*	*GAN*	*GARS1*	*GBA2*	*GBE1*
GCH1	*GDAP1*	*GGNBP2*	*GJA1*	*GJB1*	*GJB3*	*GJC2*
GLA	*GLE1*	*GNAS*	*GNB4*	*GORASP1*	*GPX3*	*GRB14*
GRN	*GSN*	*GTF2H4*	*GTF3C2*	*HACE1*	*HADHB*	*HARS1*
HBEGF	*HENMT1*	*HFE*	*HINT1*	*HK1*	*HLA-DPB1*	*HMBS*
HNRNPA1	*HNRNPA2B1*	*HNRNPD*	*HOXD10*	*HSD17B4*	*HSPB1*	*HSPB3*
HSPB8	*HSPD1*	*IARS2*	*IBA57*	*IFIH1*	*IFNA1*	*IFRD1*
IGHMBP2	*INF2*	*ITPR2*	*JPH1*	*KARS1*	*KCNA2*	*KDM5C*
KIAA1755	*KIDINS220*	*KIF1A*	*KIF1B*	*KIF1C*	*KIF5A*	*KIFAP3*

The panel of ALS related gene tested in this study.

KLC2	*KMT2C*	*L1CAM*	*LAMB1*	*LBP*	*LIMD1*	*LITAF*
LMNA	*LMNB1*	*LPP*	*LRSAM1*	*LUM*	*LYST*	*MAD1L1*
MAG	*MAPT*	*MARS1*	*MATR3*	*MED25*	*MEN1*	*METTL22*
MFN2	*MICAL1*	*MME*	*MORC2*	*MPO*	*MPV17*	*MPZ*
MTMR2	*MTRFR*	*MTTP*	*MYH14*	*MYO3B*	*NAGLU*	*NAIP*
NCAM1	*NDRG1*	*NEFH*	*NEFL*	*NEK1*	*NETO1*	*NF1*
NFU1	*NGF*	*NIPA1*	*NLRC5*	*NRL*	*NT5C2*	*NTRK1*
NUDT1	*OGG1*	*OMA1*	*OPA1*	*OPA3*	*OPTN*	*P4HB*
PANK2	*PARK7*	*PCP4*	*PDHX*	*PDIA3*	*PDK3*	*PDYN*
PEX10	*PEX12*	*PEX2*	*PEX3*	*PEX5*	*PFKL*	*PFN1*
PGAP1	*PHYH*	*PIK3CA*	*PIK3CB*	*PIK3CD*	*PIK3CG*	*PIK3R5*
PINK1	*PLA2G4C*	*PLA2G6*	*PLEKHG5*	*PLP1*	*PML*	*PMM2*
PMP2	*PMP22*	*PNKP*	*PNPLA6*	*POLG*	*PON1*	*PON2*
PON3	*PPOX*	*PPP2R2B*	*PRDM12*	*PRKCA*	*PRNP*	*PRPH*
PRPH2	*PRPS1*	*PRX*	*PSAP*	*PSEN1*	*PSMB7*	*RAB25*
RAB3GAP2	*RAB7A*	*RAD54L*	*RAMP3*	*REEP1*	*REEP2*	*RETREG1*
RINL	*RNASEH2B*	*RNF19A*	*RRAS2*	*RTN2*	*SACS*	*SBF1*
SBF2	*SCN11A*	*SCN9A*	*SCP2*	*SCYL1*	*SEPTIN9*	*SERPINE1*
SETX	*SGPL1*	*SH3TC2*	*SIGMAR1*	*SLC12A6*	*SLC16A2*	*SLC1A2*
SLC25A15	*SLC25A19*	*SLC25A46*	*SLC2A1*	*SLC30A10*	*SLC33A1*	*SLC52A2*
SLC52A3	*SLC5A7*	*SMN1*	*SND1*	*SOD1*	*SORBS1*	*SORD*
SOX10	*SOX5*	*SPART*	*SPAST*	*SPG11*	*SPG21*	*SPG7*
SPTB	*SPTLC1*	*SPTLC2*	*SPTLC3*	*SQSTM1*	*SRCAP*	*SS18L1*
STK36	*STUB1*	*STXBP1*	*SUCLA2*	*SURF1*	*SUSD1*	*SV2A*
SYNE1	*SYT2*	*SYT9*	*TAF15*	*TAF1L*	*TARDBP*	*TBC1D24*
TBK1	*TDP1*	*TECPR2*	*TF*	*TFG*	*TH*	*THSD7B*
TIA1	*TJP1*	*TMEM126A*	*TNIP1*	*TOE1*	*TOR1A*	*TREM2*
TRIM2	*TRPM2*	*TRPM7*	*TRPV4*	*TRRAP*	*TTR*	*TUBA4A*
TUBB4A	*TYMP*	*UBE2B*	*UBQLN1*	*UBQLN2*	*UCHL1*	*UNC13A*
UNC80	*USH2A*	*USP8*	*VAMP1*	*VAPB*	*VCP*	*VEGFA*
VHL	*VPS13A*	*VPS37A*	*VPS54*	*VRK1*	*WARS1*	*WASHC5*
WDR45	*WDR48*	*WDR6*	*WNK1*	*XK*	*XRCC4*	*YARS1*
ZFP64	*ZFR*	*ZFYVE26*	*ZFYVE27*	*ZNF512B*		

Supplementary table 2. *ERBB4* nonsynonymous variants identified in ALS patients and controls with related information in the public database.

CHR:POS (GRCh37)	cDNA change	Protein change	dbSNP	Exon	Frequency in patient allele	Frequency in control allele	China Map	Gnome AD （EA）	1000G （EA）
2:213403253	c.1dupA	p.Met1Asnfs*35	Novel	Exon1	0/448	1/1812	0	0	0
2:213403250	c.5A>G	p.Lys2Arg	Novel	Exon1	0/448	1/1812	0	0	0
2:213403250	c.5A>T	p.Lys2Met	Novel	Exon1	0/448	1/1812	0	0	0
2:213403249	c.6G>T	p.Lys2Asn	Novel	Exon1	0/448	1/1812	0	0	0
2:213403247	c.8C>A	p.Pro3Gln	rs772612441	Exon1	0/448	1/1812	0	0	0
2:213403235	c.20T>C	p.Leu7Pro	Novel	Exon1	0/448	1/1812	0	0	0
2:213403233	c.22T>C	p.Trp8Arg	Novel	Exon1	0/448	1/1812	0	0	0
2:213403197	c.58G>A	p.Val20Ile	rs373308672	Exon1	0/448	2/1812	0	0	0
2:213403178	c.77A>G	p.Gln26Arg	rs749626683	Exon1	0/448	1/1812	0	0	0
2:213403176	c.79T>C	p.Ser27Pro	Novel	Exon1	0/448	1/1812	0	0	0
2:212989623	c.88G>T	p.Ala30Ser	Novel	Exon2	0/448	1/1812	0	0	0
2:212989620	c.91G>T	p.Gly31*	Novel	Exon2	0/448	1/1812	0	0	0
2:212989562	c.149G>A	p.Arg50His	rs755026855	Exon2	0/448	3/1812	0	0	0
2:212989562	c.149G>T	p.Arg50Leu	rs755026855	Exon2	0/448	1/1812	0	0	0
2:212989553	c.158A>G	p.Tyr53Cys	rs756650586	Exon2	0/448	1/1812	3/21176	1/18380	0
2:212989552	c.159T>G	p.Tyr53*	rs2079870989	Exon2	0/448	1/1812	0	0	0
2:212989548	c.163A>T	p.Asn55Tyr	Novel	Exon2	0/448	1/1812	0	0	0
2:212989545	c.166T>C	p.Cys56Arg	Novel	Exon2	0/448	1/1812	0	0	0
2:212989506	c.205G>A	p.Glu69Lys	Novel	Exon2	0/448	1/1812	0	0	0
2:212989505	c.206A>T	p.Glu69Val	Novel	Exon2	0/448	1/1812	0	0	0
2:212989503	c.208C>T	p.His70Tyr	rs1357270265	Exon2	0/448	1/1812	0	0	0
2:212989496	c.215G>A	p.Arg72Gln	rs762478821	Exon2	0/448	1/1812	0	0	0
2:212812292	c.284G>A	p.Arg95His	rs778048381	Exon3	1/448	1/1812	10/21176	8/18964	0
2:212812286	c.290T>C	p.Leu97Pro	Novel	Exon3	0/448	1/1812	0	0	0
2:212812284	c.292C>A	p.Pro98Thr	rs752223901	Exon3	0/448	1/1812	0	0	0
2:212812283	c.293C>A	p.Pro98His	Novel	Exon3	0/448	1/1812	0	0	0
2:212812268	c.308G>A	p.Arg103His	rs754487821	Exon3	0/448	1/1812	0	1/19880	0
2:212812253	c.323C>T	p.Thr108Ile	Novel	Exon3	0/448	1/1812	0	0	0
2:212812221	c.355A>T	p.Ile119Leu	Novel	Exon3	0/448	1/1812	0	0	0
2:212812167	c.409A>G	p.Lys137Glu	Novel	Exon3	0/448	1/1812	0	0	0
2:212652882	c.424delA	p.Ile142Serfs*2	rs1240937160	Exon4	0/448	1/1812	0	0	0
2:212652873	c.433G>A	p.Gly145Ser	Novel	Exon4	0/448	1/1812	0	0	0
2:212652864	c.442T>C	p.Tyr148His	rs1341604623	Exon4	0/448	1/1812	0	0	0
2:212652834	c.472G>A	p.Ala158Thr	rs375361752	Exon4	0/448	1/1812	0	0	0
2:212652827	c.479C>A	p.Thr160Asn	Novel	Exon4	0/448	1/1812	0	0	0

CHR:POS (GRCh37)	cDNA change	Protein change	dbSNP	Exon	Frequency in patient allele	Frequency in control allele	China Map	Gnome AD （EA）	1000G （EA）
2:212652804	c.502C>T	p.Arg168Trp	rs138433638	Exon4	0/448	1/1812	0	0	0
2:212652788	c.518C>A	p.Ser173Tyr	Novel	Exon4	0/448	1/1812	0	0	0
2:212652781	c.525G>T	p.Leu175Phe	Novel	Exon4	0/448	1/1812	0	0	0
2:212652774	c.532G>A	p.Val178Met	Novel	Exon4	0/448	1/1812	0	0	0
2:212652762	c.544G>T	p.Gly182Cys	Novel	Exon4	0/448	1/1812	0	0	0
2:212615424	c.562C>T	p.Arg188Cys	rs752051535	Exon5	0/448	1/1812	0	0	0
2:212615396	c.590G>A	p.Cys197Tyr	rs921054446	Exon5	0/448	2/1812	0	0	0
2:212615395	c.591C>A	p.Cys197*	Novel	Exon5	0/448	1/1812	0	0	0
2:212589908	c.634G>A	p.Val212Met	Novel	Exon6	0/448	1/1812	0	0	0
2:212589895	c.647A>G	p.Gln216Arg	Novel	Exon6	0/448	1/1812	0	0	0
2:212589886	c.656G>A	p.Gly219Asp	rs751669855	Exon6	0/448	1/1812	0	0	0
2:212589880	c.662G>T	p.Cys221Phe	Novel	Exon6	0/448	2/1812	0	0	0
2:212589875	c.667G>A	p.Gly223Arg	rs758827443	Exon6	0/448	1/1812	0	0	0
2:212589863	c.679A>G	p.Ser227Gly	Novel	Exon6	0/448	1/1812	0	0	0
2:212589836	c.706G>A	p.Gly236Arg	rs2074224383	Exon6	0/448	1/1812	0	0	0
2:212589830	c.712T>A	p.Cys238Ser	Novel	Exon6	0/448	1/1812	0	0	0
2:212589830	c.712T>C	p.Cys238Arg	Novel	Exon6	0/448	1/1812	0	0	0
2:212589824	c.718G>T	p.Gly240*	Novel	Exon6	0/448	1/1812	0	0	0
2:212589817	c.725A>T	p.Lys242Met	Novel	Exon6	0/448	1/1812	0	0	0
2:212587255	c.746G>A	p.Cys249Tyr	Novel	Exon7	0/448	1/1812	0	0	0
2:212587231	c.770C>A	p.Ala257Glu	Novel	Exon7	0/448	1/1812	0	0	0
2:212587207	c.794C>A	p.Thr265Asn	Novel	Exon7	0/448	1/1812	0	0	0
2:212587142	c.859G>A	p.Ala287Thr	Novel	Exon7	0/448	1/1812	0	0	0
2:212587141	c.860C>A	p.Ala287Glu	rs1225001174	Exon7	0/448	1/1812	0	0	0
2:212587128	c.873G>T	p.Lys291Asn	Novel	Exon7	0/448	1/1812	0	0	0
2:212578369	c.888C>A	p.Asn296Lys	rs2073790313	Exon8	0/448	1/1812	0	0	0
2:212578362	c.895G>A	p.Val299Ile	rs2073790014	Exon8	0/448	1/1812	0	0	0
2:212578344	c.913G>A	p.Val305Met	Novel	Exon8	0/448	1/1812	0	0	0
2:212578341	c.916C>T	p.Arg306Cys	rs1216559792	Exon8	0/448	2/1812	0	0	0
2:212578314	c.943G>A	p.Val315Ile	Novel	Exon8	0/448	1/1812	0	0	0
2:212578292	c.965T>A	p.Met322Lys	rs2073786487	Exon8	0/448	1/1812	0	0	0
2:212578284	c.973C>A	p.Pro325Thr	rs2073786197	Exon8	0/448	1/1812	0	0	0
2:212578279	c.978C>G	p.Cys326Trp	rs1438854685	Exon8	0/448	1/1812	0	0	0
2:212578266	c.991C>A	p.Pro331Thr	rs2073785540	Exon8	0/448	1/1812	0	0	0
2:212576892	c.1007G>T	p.Gly336Val	rs866948313	Exon9	0/448	1/1812	0	0	0
2:212576890	c.1009A>G	p.Ile337Val	rs1184477882	Exon9	0/448	1/1812	0	0	0
2:212576886	c.1013G>A	p.Gly338Asp	Novel	Exon9	0/448	1/1812	0	0	0

CHR:POS (GRCh37)	cDNA change	Protein change	dbSNP	Exon	Frequency in patient allele	Frequency in control allele	China Map	Gnome AD（EA）	1000G （EA）
2:212576872	c.1027A>C	p.Met343Leu	rs2073724646	Exon9	0/448	1/1812	0	0	0
2:212576870	c.1029G>A	p.Met343Ile	Novel	Exon9	0/448	1/1812	0	0	0
2:212576850	c.1049C>A	p.Ser350Tyr	Novel	Exon9	0/448	1/1812	0	0	0
2:212576842	c.1057A>G	p.Ile353Val	rs921053875	Exon9	0/448	1/1812	0	0	0
2:212576837	c.1062C>A	p.Asp354Glu	rs2073723228	Exon9	0/448	1/1812	0	0	0
2:212576828	c.1071A>G	p.Ile357Met	rs767369850	Exon9	0/448	1/1812	0	0	0
2:212576808	c.1091G>T	p.Gly364Val	Novel	Exon9	0/448	1/1812	0	0	0
2:212576778	c.1121A>G	p.His374Arg	rs2073721432	Exon9	0/448	1/1812	0	0	0
2:212576777	c.1122T>G	p.His374Gln	rs76603692	Exon9	0/448	1/1812	0	0	0
2:212570100	c.1141A>G	p.Ile381Val	rs760614876	Exon10	0/448	1/1812	0	0	0
2:212570070	c.1171G>A	p.Val391Ile	rs745408040	Exon10	0/448	1/1812	1/21176	1/18394	0
2:212568905	c.1213C>T	p.Gln405*	Novel	Exon11	0/448	1/1812	0	0	0
2:212568901	c.1217C>A	p.Ser406*	Novel	Exon11	0/448	1/1812	0	0	0
2:212568898	c.1220G>A	p.Trp407*	Novel	Exon11	0/448	1/1812	0	0	0
2:212568892	c.1226C>A	p.Pro409Gln	rs868543170	Exon11	0/448	1/1812	0	0	0
2:212568886	c.1232T>C	p.Met411Thr	Novel	Exon11	0/448	1/1812	0	0	0
2:212568883	c.1235C>T	p.Thr412Ile	Novel	Exon11	0/448	2/1812	0	0	0
2:212568871	c.1247T>G	p.Val416Gly	Novel	Exon11	0/448	1/1812	0	0	0
2:212568859	c.1259T>C	p.Leu420Pro	Novel	Exon11	0/448	1/1812	0	0	0
2:212568847	c.1271G>T	p.Gly424Val	Novel	Exon11	0/448	1/1812	0	0	0
2:212568833	c.1285T>C	p.Tyr429His	Novel	Exon11	0/448	1/1812	0	0	0
2:212566887	c.1294C>A	p.Leu432Met	Novel	Exon12	0/448	1/1812	0	0	0
2:212566879	c.1302G>T	p.Leu434Phe	rs1197875570	Exon12	0/448	1/1812	0	0	0
2:212566869	c.1312A>C	p.Lys438Gln	Novel	Exon12	0/448	1/1812	0	0	0
2:212566866	c.1315C>T	p.Gln439*	Novel	Exon12	0/448	1/1812	0	0	0
2:212566832	c.1349T>C	p.Leu450Pro	Novel	Exon12	0/448	1/1812	0	0	0
2:212566818	c.1363G>T	p.Ala455Ser	rs762866612	Exon12	0/448	1/1812	0	0	0
2:212566811	c.1370A>C	p.Asn457Thr	Novel	Exon12	0/448	1/1812	0	0	0
2:212566791	c.1390A>G	p.Ser464Gly	Novel	Exon12	0/448	1/1812	0	0	0
2:212566790	c.1391G>A	p.Ser464Asn	Novel	Exon12	0/448	1/1812	0	0	0
2:212566789	c.1392C>A	p.Ser464Arg	rs1436708940	Exon12	0/448	1/1812	0	0	0
2:212566772	c.1409A>G	p.His470Arg	Novel	Exon12	0/448	1/1812	0	0	0
2:212566694	c.1487G>A	p.Cys496Tyr	Novel	Exon12	0/448	1/1812	0	0	0
2:212566692	c.1489A>T	p.Thr497Ser	Novel	Exon12	0/448	1/1812	0	0	0
2:212543888	c.1511A>T	p.Asn504Ile	rs2072233597	Exon13	0/448	1/1812	0	0	0
2:212543871	c.1528G>A	p.Asp510Asn	rs1361402584	Exon13	0/448	1/1812	0	2/18388	0

CHR:POS (GRCh37)	cDNA change	Protein change	dbSNP	Exon	Frequency in patient allele	Frequency in control allele	China Map	Gnome AD（EA）	1000G （EA）
2:212543868	c.1531G>A	p.Gly511Ser	Novel	Exon13	0/448	1/1812	0	0	0
2:212543867	c.1532G>A	p.Gly511Asp	Novel	Exon13	0/448	1/1812	0	0	0
2:212543847	c.1552G>A	p.Asp518Asn	rs2072232112	Exon13	0/448	1/1812	0	0	0
2:212543845	c.1554C>A	p.Asp518Glu	rs1471389448	Exon13	0/448	1/1812	0	0	0
2:212543844	c.1555C>A	p.Gln519Lys	Novel	Exon13	0/448	1/1812	0	0	0
2:212543828	c.1571G>A	p.Arg524His	rs371593463	Exon13	0/448	1/1812	0	1/19936	0
2:212543802	c.1597A>T	p.Ile533Leu	rs780678450	Exon13	0/448	1/1812	0	0	0
2:212543789	c.1610A>G	p.Asn537Ser	rs879211730	Exon13	0/448	1/1812	0	0	0
2:212543780	c.1619A>T	p.Asp540Val	rs878906416	Exon13	0/448	1/1812	0	0	0
2:212537981	c.1624G>A	p.Glu542Lys	rs535202189	Exon14	0/448	2/1812	7/21176	16/18390	0.001
2:212537975	c.1630C>T	p.Arg544Trp	rs267599192	Exon14	0/448	1/1812	0	0	0
2:212537959	c.1646G>A	p.Gly549Asp	rs772962110	Exon14	0/448	2/1812	0	0	0
2:212537945	c.1660G>A	p.Glu554Lys	Novel	Exon14	0/448	1/1812	0	0	0
2:212537944	c.1661A>G	p.Glu554Gly	rs1322856972	Exon14	0/448	1/1812	0	0	0
2:212537893	c.1712G>A	p.Gly571Glu	rs878861872	Exon14	0/448	1/1812	0	0	0
2:212537891	c.1714C>T	p.Pro572Ser	rs998449284	Exon14	0/448	1/1812	0	0	0
2:212530202	c.1717G>T	p.Gly573Cys	rs750966942	Exon15	0/448	1/1812	0	0	0
2:212530121	c.1798G>A	p.Ala600Thr	rs1229347539	Exon15	0/448	1/1812	0	0	0
2:212530118	c.1801A>G	p.Asn601Asp	rs1229347539	Exon15	0/448	1/1812	0	0	0
2:212530085	c.1834C>T	p.Arg612Trp	rs561997094	Exon15	0/448	1/1812	7/21176	8/18394	0
2:212530063	c.1856C>T	p.Pro619Leu	Novel	Exon15	0/448	1/1812	0	0	0
2:212522551	c.1874G>A	p.Cys625Tyr	rs894409474	Exon16	0/448	1/1812	0	0	0
2:212522549	c.1876A>G	p.Asn626Asp	Novel	Exon16	0/448	1/1812	0	0	0
2:212522546	c.1879G>A	p.Gly627Ser	rs767530108	Exon16	0/448	1/1812	0	0	0
2:212522540	c.1885A>G	p.Thr629Ala	rs761677465	Exon16	0/448	1/1812	0	0	0
2:212522531	c.1894G>A	p.Asp632Asn	rs528082386	Exon16	0/448	1/1812	1/21176	0	0
2:212522515	c.1910C>A	p.Pro637Gln	Novel	Exon16	0/448	1/1812	0	0	0
2:212522507	c.1918G>T	p.Gly640Cys	Novel	Exon16	0/448	1/1812	0	0	0
2:212522501	c.1924T>C	p.Ser642Pro	Novel	Exon16	0/448	1/1812	0	0	0
2:212522497	c.1928C>A	p.Thr643Asn	rs779321314	Exon16	0/448	1/1812	0	0	0
2:212522482	c.1943C>G	p.Ala648Gly	Novel	Exon16	0/448	1/1812	0	0	0
2:212522479	c.1946G>C	p.Arg649Thr	rs978290380	Exon16	0/448	1/1812	0	0	0
2:212495314	c.1952C>T	p.Pro651Leu	rs1337377088	Exon17	0/448	1/1812	0	0	0
2:212495305	c.1961C>T	p.Ala654Val	Novel	Exon17	0/448	1/1812	0	0	0
2:212530126	c.1793A>T	p.Gln598Leu	Novel	Exon15	0/448	1/1812	0	0	0

**CHR:POS (GRCh37)**	**cDNA change**	**Protein change**	**dbSNP**	**Exon**	**Frequency in patient allele**	**Frequency in control allele**	**China Map**	**Gnome AD（EA）**	**1000G （EA）**
2:212495294	c.1972A>T	p.Ile658Phe	rs190654033	Exon17	5/448	20/1812	123/21176	68/19952	0.003
2:212495270	c.1996A>G	p.Ile666Val	rs370786638	Exon17	0/448	1/1812	0	0	0
2:212495263	c.2003G>A	p.Gly668Asp	rs1449246579	Exon17	0/448	1/1812	0	0	0
2:212495251	c.2015C>T	p.Ala672Val	Novel	Exon17	0/448	1/1812	0	0	0
2:212488754	c.2095A>G	p.Thr699Ala	rs774321172	Exon18	0/448	1/1812	0	0	0
2:212488720	c.2129T>G	p.Leu710Arg	Novel	Exon18	0/448	1/1812	0	0	0
2:212488718	c.2131C>A	p.Arg711Ser	rs267599191	Exon18	0/448	1/1812	0	0	0
2:212488717	c.2132G>A	p.Arg711His	rs1269407579	Exon18	0/448	1/1812	0	0	0
2:212488691	c.2158A>T	p.Arg720Trp	rs868205149	Exon18	0/448	1/1812	0	0	0
2:212488683	c.2166A>T	p.Lys722Asn	rs1422888414	Exon18	0/448	1/1812	0	0	0
2:212488682	c.2167G>A	p.Val723Ile	rs1365046282	Exon18	0/448	1/1812	0	0	0
2:212488666	c.2183C>T	p.Ala728Val	Novel	Exon18	0/448	1/1812	2/21176	0	0
2:212488657	c.2192C>T	p.Thr731Met	rs374970657	Exon18	0/448	1/1812	1/21176	0	0
2:212488655	c.2194G>A	p.Val732Ile	Novel	Exon18	0/448	1/1812	0	0	0
2:212488652	c.2197T>C	p.Tyr733His	Novel	Exon18	0/448	1/1812	0	0	0
2:212483991	c.2212G>A	p.Val738Ile	rs1379834725	Exon19	0/448	1/1812	0	0	0
2:212483973	c.2230G>A	p.Val744Met	Novel	Exon19	0/448	1/1812	0	0	0
2:212483968	c.2235G>T	p.Lys745Asn	Novel	Exon19	0/448	1/1812	0	0	0
2:212483964	c.2239C>A	p.Pro747Thr	Novel	Exon19	0/448	1/1812	0	0	0
2:212483940	c.2263G>A	p.Glu755Lys	Novel	Exon19	0/448	1/1812	0	0	0
2:212483939	c.2264A>G	p.Glu755Gly	rs2069502811	Exon19	0/448	1/1812	0	0	0
2:212483938	c.2265G>T	p.Glu755Asp	rs1185506318	Exon19	0/448	1/1812	0	0	0
2:212483933	c.2270C>A	p.Thr757Asn	Novel	Exon19	0/448	1/1812	0	0	0
2:212483928	c.2275C>T	p.Pro759Ser	Novel	Exon19	0/448	1/1812	0	0	0
2:212483925	c.2278A>G	p.Lys760Glu	Novel	Exon19	0/448	1/1812	0	0	0
2:212483923	c.2280G>T	p.Lys760Asn	rs1261603409	Exon19	0/448	1/1812	0	0	0
2:212483919	c.2284A>G	p.Asn762Asp	Novel	Exon19	0/448	1/1812	0	0	0
2:212483904	c.2299G>A	p.Asp767Asn	Novel	Exon19	0/448	1/1812	0	0	0
2:212426786	c.2329C>A	p.His777Asn	rs866491191	Exon20	0/448	1/1812	0	0	0
2:212426758	c.2357T>C	p.Val786Ala	Novel	Exon20	0/448	1/1812	0	0	0
2:212426755	c.2360G>T	p.Cys787Phe	Novel	Exon20	0/448	1/1812	0	0	0
2:212426720	c.2395A>G	p.Met799Val	rs753682457	Exon20	0/448	1/1812	0	1/18392	0
2:212426714	c.2401C>A	p.His801Asn	rs1217205766	Exon20	0/448	1/1812	0	0	0
2:212426713	c.2402A>G	p.His801Arg	rs1420072649	Exon20	0/448	1/1812	0	0	0
2:212426679	c.2436G>T	p.Lys812Asn	rs2067405713	Exon20	0/448	1/1812	0	0	0

CHR:POS (GRCh37)	cDNA change	Protein change	dbSNP	Exon	Frequency in patient allele	Frequency in control allele	China Map	Gnome AD（EA）	1000G （EA）
2:212295817	c.2496G>A	p.Met832Ile	rs867157034	Exon21	0/448	1/1812	0	0	0
2:212295807	c.2506G>T	p.Glu836*	Novel	Exon21	0/448	1/1812	0	0	0
2:212295795	c.2518G>A	p.Val840Ile	rs369248674	Exon21	0/448	1/1812	0	0	0
2:212295779	c.2534C>T	p.Ala845Val	Novel	Exon21	0/448	1/1812	0	0	0
2:212295776	c.2537C>A	p.Ala846Asp	rs758104381	Exon21	0/448	1/1812	0	0	0
2:212295767	c.2546T>C	p.Val849Ala	Novel	Exon21	0/448	1/1812	0	0	0
2:212295767	c.2546T>G	p.Val849Gly	Novel	Exon21	0/448	1/1812	0	0	0
2:212295761	c.2552T>C	p.Val851Ala	Novel	Exon21	0/448	1/1812	0	0	0
2:212295744	c.2569G>T	p.Val857Leu	Novel	Exon21	0/448	1/1812	0	0	0
2:212295723	c.2590C>A	p.Leu864Ile	rs1349871841	Exon21	0/448	1/1812	0	0	0
2:212295714	c.2599C>A	p.Leu867Ile	Novel	Exon21	0/448	1/1812	0	0	0
2:212295701	c.2612A>G	p.Asp871Gly	rs1480577684	Exon21	0/448	1/1812	0	0	0
2:212295694	c.2619delA	p.Glu874Serfs*40	rs747015469	Exon21	0/448	1/1812	0	0	0
2:212295678	c.2635G>A	p.Gly879Arg	rs1198974786	Exon21	0/448	1/1812	0	0	0
2:212293206	c.2646G>T	p.Met882Ile	rs771843850	Exon22	0/448	1/1812	0	0	0
2:212293205	c.2647C>A	p.Pro883Thr	rs747975697	Exon22	0/448	1/1812	0	0	0
2:212293205	c.2647C>T	p.Pro883Ser	rs747975697	Exon22	0/448	1/1812	0	0	0
2:212293194	c.2658G>T	p.Trp886Cys	Novel	Exon22	0/448	2/1812	0	0	0
2:212293193	c.2659A>G	p.Met887Val	rs2063682033	Exon22	0/448	1/1812	0	0	0
2:212293191	c.2661G>A	p.Met887Ile	rs754748646	Exon22	0/448	1/1812	1/21176	0	0
2:212293190	c.2662G>T	p.Ala888Ser	Novel	Exon22	0/448	1/1812	0	0	0
2:212293186	c.2666T>C	p.Leu889Pro	rs2063681823	Exon22	0/448	1/1812	0	0	0
2:212293180	c.2672G>A	p.Cys891Tyr	rs181319801	Exon22	0/448	1/1812	0	1/18338	0.001
2:212293174	c.2678A>G	p.His893Arg	rs1228208231	Exon22	0/448	1/1812	0	0	0
2:212293171	c.2681A>G	p.Tyr894Cys	Novel	Exon22	0/448	1/1812	0	0	0
2:212293157	c.2695C>T	p.His899Tyr	Novel	Exon22	0/448	1/1812	0	0	0
2:212293137	c.2715C>A	p.Ser905Arg	rs758894552	Exon22	0/448	1/1812	0	0	0
2:212293136	c.2716T>C	p.Tyr906His	Novel	Exon22	0/448	1/1812	0	0	0
2:212289018	c.2728A>G	p.Ile910Val	rs754195521	Exon23	0/448	1/1812	1/21176	1/19940	0
2:212289017	c.2729T>C	p.Ile910Thr	Novel	Exon23	0/448	1/1812	1/21176	0	0
2:212288972	c.2774C>T	p.Pro925Leu	Novel	Exon23	0/448	1/1812	0	0	0
2:212288969	c.2777C>A	p.Thr926Lys	rs148791072	Exon23	0/448	1/1812	1/21176	0	0.001
2:212288969	c.2777C>T	p.Thr926Met	rs148791072	Exon23	0/448	2/1812	1/21176	10/18384	0.001
2:212288967	c.2779C>T	p.Arg927*	rs537458255	Exon23	0/448	1/1812	0	0	0
2:212288964	c.2782G>A	p.Glu928Lys	rs2063576455	Exon23	0/448	1/1812	0	0	0

CHR:POS (GRCh37)	cDNA change	Protein change	dbSNP	Exon	Frequency in patient allele	Frequency in control allele	China Map	Gnome AD（EA）	1000G （EA）
2:212288934	c.2812C>T	p.Arg938Cys	rs2063575508	Exon23	0/448	1/1812	0	0	0
2:212288921	c.2825C>A	p.Pro942His	Novel	Exon23	0/448	2/1812	0	0	0
2:212288916	c.2830A>G	p.Ile944Val	rs1435419609	Exon23	0/448	1/1812	0	3/18392	0
2:212288886	c.2860G>A	p.Val954Ile	rs758295583	Exon23	0/448	1/1812	0	0	0
2:212286827	c.2869T>C	p.Trp957Arg	Novel	Exon24	0/448	1/1812	0	0	0
2:212286822	c.2874G>A	p.Met958Ile	Novel	Exon24	0/448	1/1812	0	0	0
2:212286815	c.2881G>A	p.Ala961Thr	Novel	Exon24	0/448	1/1812	0	0	0
2:212286808	c.2888G>A	p.Ser963Asn	rs1172120091	Exon24	0/448	1/1812	0	0	0
2:212286793	c.2903A>G	p.Lys968Arg	Novel	Exon24	0/448	1/1812	0	0	0
2:212286791	c.2905G>T	p.Glu969*	Novel	Exon24	0/448	1/1812	0	0	0
2:212286784	c.2912C>A	p.Ala971Asp	Novel	Exon24	0/448	1/1812	0	0	0
2:212286772	c.2924C>T	p.Ser975Leu	rs2063524718	Exon24	0/448	1/1812	0	0	0
2:212286763	c.2933C>T	p.Ala978Val	Novel	Exon24	0/448	1/1812	0	0	0
2:212286761	c.2935C>G	p.Arg979Gly	rs574197848	Exon24	0/448	2/1812	8/21176	4/18390	0
2:212286760	c.2936G>A	p.Arg979Gln	rs763644012	Exon24	0/448	3/1812	5/21176	14/19946	0
2:212286758	c.2938G>A	p.Asp980Asn	Novel	Exon24	0/448	1/1812	0	0	0
2:212286758	c.2938G>T	p.Asp980Tyr	Novel	Exon24	0/448	1/1812	0	0	0
2:212286756	c.2940C>A	p.Asp980Glu	Novel	Exon24	0/448	1/1812	0	0	0
2:212286751	c.2945A>G	p.Gln982Arg	Novel	Exon24	0/448	1/1812	0	0	0
2:212286737	c.2959A>G	p.Ile987Val	Novel	Exon24	0/448	1/1812	0	0	0
2:212286734	c.2962C>T	p.Gln988*	rs2063524008	Exon24	0/448	1/1812	0	0	0
2:212286732	c.2964G>T	p.Gln988His	rs78668383	Exon24	0/448	1/1812	0	0	0
2:212285333	c.2968G>C	p.Asp990His	Novel	Exon25	0/448	1/1812	0	0	0
2:212285330	c.2971G>A	p.Asp991Asn	Novel	Exon25	0/448	1/1812	0	0	0
2:212285299	c.3002G>A	p.Ser1001Asn	rs766321838	Exon25	0/448	1/1812	0	0	0
2:212285285	c.3016A>T	p.Asn1006Tyr	Novel	Exon25	0/448	1/1812	0	0	0
2:212285268	c.3033G>T	p.Glu1011Asp	rs1218019126	Exon25	0/448	1/1812	0	0	0
2:212285245	c.3056C>A	p.Ala1019Asp	Novel	Exon25	0/448	1/1812	0	0	0
2:212285241	c.3060G>T	p.Glu1020Asp	Novel	Exon25	0/448	1/1812	2/21176	0	0
2:212285236	c.3065A>G	p.Tyr1022Cys	Novel	Exon25	0/448	1/1812	0	0	0
2:212285224	c.3077A>G	p.Gln1026Arg	Novel	Exon25	0/448	1/1812	0	0	0
2:212285222	c.3079G>A	p.Ala1027Thr	rs2063491832	Exon25	0/448	1/1812	0	0	0
2:212285210	c.3091C>A	p.Pro1031Thr	Novel	Exon25	0/448	1/1812	0	0	0
2:212285209	c.3092C>A	p.Pro1031Gln	rs369858826	Exon25	0/448	1/1812	0	0	0
2:212285185	c.3116C>A	p.Ala1039Glu	Novel	Exon25	0/448	1/1812	0	0	0

CHR:POS (GRCh37)	cDNA change	Protein change	dbSNP	Exon	Frequency in patient allele	Frequency in control allele	China Map	Gnome AD（EA）	1000G （EA）
2:212252702	c.3151A>G	p.Ser1051Gly	Novel	Exon26	0/448	1/1812	0	0	0
2:212252701	c.3152G>T	p.Ser1051Ile	Novel	Exon26	0/448	1/1812	0	0	0
2:212252687	c.3166T>A	p.Tyr1056Asn	rs533283389	Exon26	0/448	1/1812	0	0	0
2:212252681	c.3172C>A	p.Pro1058Thr	rs2062721597	Exon26	0/448	1/1812	0	0	0
2:212252680	c.3173C>A	p.Pro1058His	rs2062721540	Exon26	0/448	1/1812	0	0	0
2:212252677	c.3176T>C	p.Met1059Thr	rs373685875	Exon26	0/448	1/1812	0	0	0
2:212252676	c.3177G>T	p.Met1059Ile	Novel	Exon26	0/448	1/1812	0	0	0
2:212251853	c.3206G>A	p.Gly1069Glu	rs1235299802	Exon27	0/448	1/1812	0	0	0
2:212251844	c.3215C>G	p.Ala1072Gly	Novel	Exon27	0/448	1/1812	0	0	0
2:212251779	c.3280G>A	p.Ala1094Thr	rs2062700383	Exon27	0/448	1/1812	0	0	0
2:212251752	c.3307G>T	p.Asp1103Tyr	Novel	Exon27	0/448	1/1812	1/21176	1/18394	0
2:212251728	c.3331C>A	p.Leu1111Ile	Novel	Exon27	0/448	1/1812	0	0	0
2:212251712	c.3347C>T	p.Ala1116Val	Novel	Exon27	0/448	1/1812	0	0	0
2:212251707	c.3352C>T	p.His1118Tyr	Novel	Exon27	0/448	1/1812	0	0	0
2:212251695	c.3364G>A	p.Asp1122Asn	rs747886025	Exon27	0/448	1/1812	0	0	0
2:212251659	c.3400G>A	p.Val1134Met	rs139785964	Exon27	0/448	1/1812	0	3/19954	0
2:212251645	c.3414A>C	p.Glu1138Asp	rs1260865838	Exon27	0/448	1/1812	0	0	0
2:212251622	c.3437A>T	p.Asp1146Val	rs768479400	Exon27	0/448	1/1812	1/21176	1/18394	0
2:212251620	c.3439G>C	p.Glu1147Gln	rs2062695832	Exon27	0/448	1/1812	0	0	0
2:212251610	c.3449A>G	p.Tyr1150Cys	rs1187421880	Exon27	0/448	1/1812	0	0	0
2:212251609	c.3450C>A	p.Tyr1150*	Novel	Exon27	0/448	1/1812	0	0	0
2:212251583	c.3476A>G	p.Lys1159Arg	Novel	Exon27	0/448	1/1812	0	0	0
2:212248773	c.3494C>A	p.Pro1165Gln	rs202042335	Exon28	0/448	1/1812	0	0	0
2:212248759	c.3508C>A	p.Pro1170Thr	Novel	Exon28	0/448	1/1812	0	0	0
2:212248758	c.3509C>A	p.Pro1170His	Novel	Exon28	0/448	1/1812	0	0	0
2:212248564	c.3703A>G	p.Lys1235Glu	Novel	Exon28	0/448	1/1812	0	0	0
2:212248553	c.3714C>A	p.Asp1238Glu	Novel	Exon28	0/448	1/1812	0	0	0
2:212248498	c.3769G>C	p.Asp1257His	rs766270456	Exon28	1/448	1/1812	3/21176	13/18376	o
2:212248377	c.3890C>A	p.Pro1297Gln	rs751834116	Exon28	0/448	1/1812	0	o	0
2:212248363	c.3904A>G	p.Arg1302Gly	rs1466199554	Exon28	0/448	1/1812	0	0	0

Key: dbSNP, The Single Nucleotide Polymorphism Database; gnomAD, Genome Aggregation Database; ChinaMAP, China Metabolic Analytics Project.

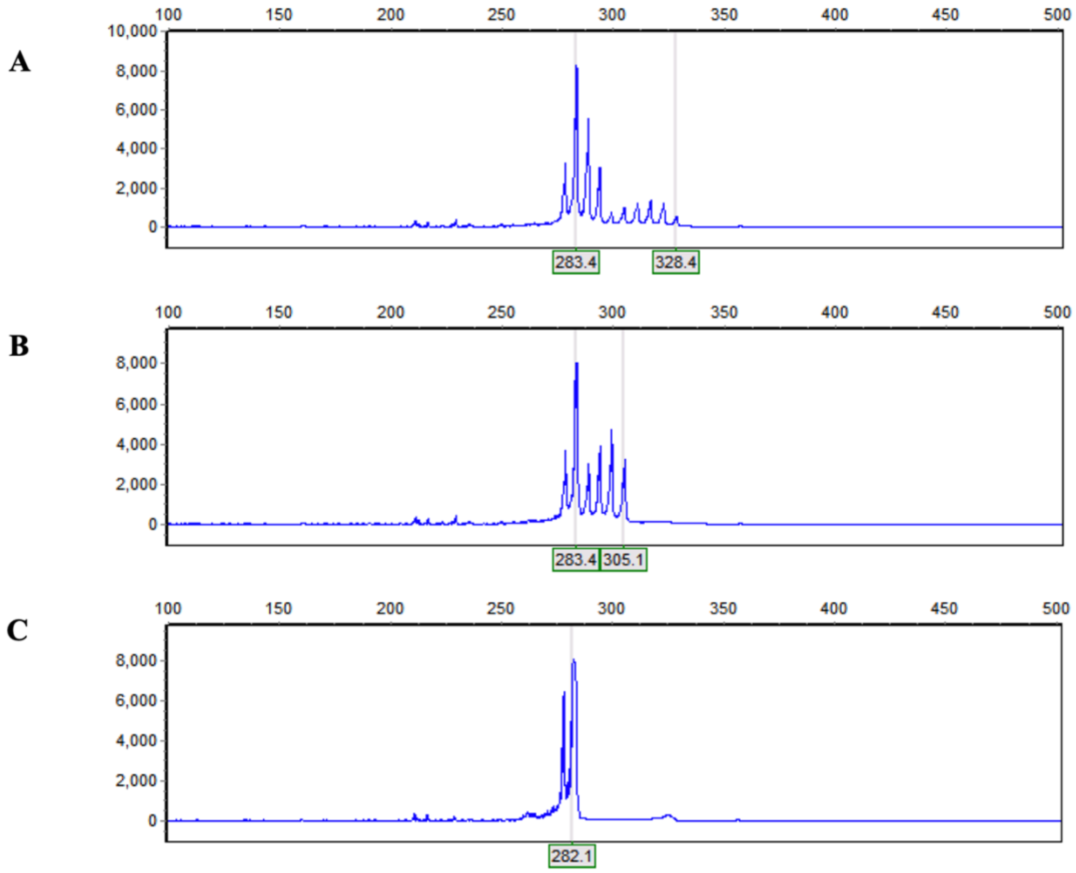

Supplementary figure 1. The chromatograph of repeat primed PCR for *C9orf72* in the three patients who carrying likely pathogenic variants. (A) The chromatograph of repeat primed PCR for *C9orf72* in the three patients who carrying p.Arg106His. (B) The chromatograph of repeat primed PCR for *C9orf72* in the three patients who carrying p.Gln164Pro. (C) The chromatograph of repeat primed PCR for *C9orf72* in the three patients who carrying p.Val212Leu. X-axis represents fragment length (nt)and Y-axis represents fluorescence signal intensity.
